# Draft Genome Sequence of *Streptomyces* sp. Strain G-5, Isolated from Marine Sponge, Kochi Prefecture, Japan

**DOI:** 10.1128/mra.01054-22

**Published:** 2022-12-14

**Authors:** Yuta Matsubara, Tetsuya Sakurai, Dana Ulanova

**Affiliations:** a Graduate School of Integrated Arts and Sciences, Department of Agriculture and Marine Science, Kochi University, Nankoku, Kochi, Japan; b Department of Marine Resource Science, Faculty of Agriculture and Marine Science, Kochi University, Nankoku, Kochi, Japan; c Center for Advanced Marine Core Research, Kochi University, Nankoku, Kochi, Japan; Montana State University

## Abstract

Actinomycetes isolated from the marine environment often require the presence of seawater for their growth and/or morphological development. Here, we report the isolation and genome sequencing of marine sponge-derived *Streptomyces* sp. strain G-5 with such a seawater requirement.

## ANNOUNCEMENT

Actinomycetes, especially *Streptomyces* spp., are known as bacteria that produce a range of bioactive compounds (1, 2). They are present in all environments and each environment influences their metabolic processes (3, 4). Many marine actinomycetes show seawater-dependent growth, and the genome-based understanding of such a requirement is important from evolutionary, ecological, and biotechnological points of view (5, 6). This study reports the isolation and genome sequencing of marine sponge-derived *Streptomyces* sp. strain G-5 which shows optimal growth in seawater-containing medium.

A marine sponge (Demospongiae) sample was collected by scuba diving offshore Kochi Prefecture, Japan (33°25′27.25 N, 133°27′29.04 E, water depth of 10 to ~15m). The sample surface was washed by sterile seawater and removed. The sponge internal part was crushed by using a pestle and mortar. The obtained mixture was inoculated on AMM agar medium (7) with natural seawater, cycloheximide (100 mg · L^−1^), and rifampicin (5 mg · L^−1^). The actinobacterium-like colony was observed after 14 days at 28°C. The isolate required seawater for optimal growth and morphological development.

For genomic DNA extraction, the isolate was cultured in AMM liquid medium containing seawater (3 days, 28°C, and 160 rpm) and genomic DNA was extracted using the genomic DNA buffer set and Genomic-tip 20/G (both Qiagen) according to the manufacturer’s protocol. The DNA quantity and quality were evaluated by a Qubit 3.0 fluorometer (Thermo Fisher) and NanoVue Plus spectrophotometer (GE Healthcare).

The library preparation and sequencing were performed by Macrogen Japan using the TruSeq DNA PCR-free (350) kit and NovaSeq 6000 platform (2 × 150-bp paired-end reads) (Illumina). A total of 4,613,809 reads were generated. The raw reads were trimmed using Trimmomatic v0.39 (8) to remove adapter and low-quality sequences. The 4,200,000 obtained reads were assembled by the SPAdes assembler v3.15.5 (9). The genome annotation was generated by the NCBI Prokaryotic Genome Annotation Pipeline (PGAP) (10). The prediction of biosynthetic gene clusters for bioactive compounds (BGCs) was done by antiSMASH v6.1.1 (11). All tools except for Trimmomatic were run with default parameters. Trimmomatic was run with LEADING:10 TRAILING:10 SLIDINGWINDOW:4:20 MINLEN:75 options.

The draft genome of *Streptomyces* sp. strain G-5 has a total length of 6,194,373 bp and is distributed in 35 contigs, with the largest contig being 1,118,529 bp long. The average coverage was approximately 200×; the *N*_50_ value for assembly was 605,269 bp, and the GC content was 72.0%. The annotation identified 5,716 total genes; 5,645 total coding sequences (CDSs); 58 tRNAs; and 3 each of the partial 5S, 16S, and 23S rRNAs. The 16S rRNA gene sequence BLASTn analysis (nucleotide collection database) (12) revealed that the isolate shared 100% identity with Streptomyces xiamenensis strain 318 (GenBank CP009922.3). The presence of 24 full or partial BGCs was predicted.

This study adds a new strain of marine sponge-derived *Streptomyces* and contributes to the knowledge on the genomic diversity of actinomycetes in the marine environment.

### Data availability.

This whole-genome shotgun project has been deposited at DDBJ/ENA/GenBank under the accession JAOQAQ000000000. The version described in this paper is the first version, JAOQAQ010000000. The raw reads are available in the Sequence Read Archive (SRA) under accession number SRX17468437.
